# ECG Data Analysis with Denoising Approach and Customized CNNs

**DOI:** 10.3390/s22051928

**Published:** 2022-03-01

**Authors:** Abhinav Mishra, Ganapathiraju Dharahas, Shilpa Gite, Ketan Kotecha, Deepika Koundal, Atef Zaguia, Manjit Kaur, Heung-No Lee

**Affiliations:** 1 Symbiosis Institute of Technology, Pune 412115, India; abhinav.mishra.btech2019@sitpune.edu.in (A.M.); ganapathiraju.dharahas.btech2019@sitpune.edu.in (G.D.); shilpa.gite@sitpune.edu.in (S.G.); 2 Symbiosis Centre for Applied AI, Symbiosis International (Deemed) University, Pune 412115, India; head@scaai.siu.edu.in; 3 Department of Systemics, School of Computer Science, University of Petroleum and Energy Studies, Dehradun 248007, India; dkoundal@ddn.upes.ac.in; 4 Department of Computer Science, College of Computers and Information Technology, Taif University, P.O. Box 11099, Taif 21944, Saudi Arabia; zaguia.atef@tu.edu.sa; 5 School of Electrical Engineering and Computer Science, Gwangju Institute of Science and Technology, Gwangju 61005, Korea; manjitbhinder8@gmail.com

**Keywords:** filters, denoising, customized CCNNs, median filters, Gaussian filter, wavelet filters, moving average filters, Savitzky–Golay filters, low-pass Butterworth filters

## Abstract

In the last decade, the proactive diagnosis of diseases with artificial intelligence and its aligned technologies has been an exciting and fruitful area. One of the areas in medical care where constant monitoring is required is cardiovascular diseases. Arrhythmia, one of the cardiovascular diseases, is generally diagnosed by doctors using Electrocardiography (ECG), which records the heart’s rhythm and electrical activity. The use of neural networks has been extensively adopted to identify abnormalities in the last few years. It is found that the probability of detecting arrhythmia increases if the denoised signal is used rather than the raw input signal. This paper compares six filters implemented on ECG signals to improve classification accuracy. Custom convolutional neural networks (CCNNs) are designed to filter ECG data. Extensive experiments are drawn by considering the six ECG filters and the proposed custom CCNN models. Comparative analysis reveals that the proposed models outperform the competitive models in various performance metrics.

## 1. Introduction

Medical assistance with technology has been increasing rapidly over the last few years. It is an efficient way to treat and monitor patients who require constant medical support. Cardiovascular diseases are primal diseases that humans have been facing for many years. The heart is a vital organ in the body, and identification and diagnosis of heart diseases is critical. These diseases are caused by interruption of oxygen supply to heart muscles due to blockages, resulting in muscle death. Reports cite that 17.9 million people die of arrhythmia each year, estimated at 32% of all deaths [1]. With changing lifestyles and food habits worldwide, all the age groups are now being affected by these diseases, and the pattern of identification became random. Generally, arrhythmia is identified by ECG, which records electricity flowing through the heart [2]. ECG must be studied deeply to identify trends in the graph to predict arrhythmia. A person that is affected by the disease and is prone to it needs continuous observation by ECG.

The scarcity of trained medical professionals in lower-middle- and low-income countries is prevalent, especially in rural areas [3]. Even continuous monitoring of heart disease by an individual is so difficult. With ECG data, arrhythmia identification can be made using computer-aided diagnosis systems [2]. Recent advancements in the computer field, such as enhanced GPUs and computer vision and the availability of datasets, have made it possible to identify trends in ECG which indicate these diseases [4]. The use of neural networks has made it fast, easy, and cheap to give accurate results. Many countries worldwide are now focusing on the healthcare sector and giving importance to its annual budget, but it can still not fulfill society’s aspirations. Furthermore, the lack of health workers puts pressure on existing members, which can be solved by automating some tasks and making machines think using deep learning [5].

Solutions with deep learning and machine learning have been suggested for many medical applications, especially those requiring immediate attention, constant monitoring, and patients in critical condition. Deep learning helps in image classification and audio analysis, now widely used in the research community. The significant contributions of this work can be stated as follows:

1. Denoising the raw ECG data to extract accurate data.
2. The use of custom convolution neural networks produced 94% and 93% accuracy to analyze the signals and observe the patterns.

The novelty of this paper lies in the comprehensive comparison of six (6) widely used filters for denoising waveforms, finding out the filter which performs best on ECG data. Then, applying the filter onto the data and using the denoised information to train and test the three (3) custom convolutional neural networks (CCNNs) built using a combination of different types of layers and corresponding parameters. The advantages and disadvantages of implementing custom convolutional neural networks (CCNNs) on ECG data are widely discussed.

The paper is organized as follows. Section 2 states the literature review. Section 3 describes the data set and the filters used for denoising. Section 4 represents the architecture of the custom convolution neural networks used. Section 5 presents the results and discussion. Finally, Section 6 concludes the findings of the implementation and future scope of the work.

## 2. Related Work

Detection of arrhythmia is an important and challenging task. ECG signals are analyzed to identify the patterns and detect the insights. Almahamdy et al. [6] used different denoising techniques to filter out and get a pure ECG signal. Hilbert transform is one of the efficient methods for denoising the signal. Sabut et al. [7] used this method and adaptive filters for filtering out the input ECG. Altan et al. [8] applied Hilbert transform on the initial data to identify the complexes, and the results obtained were used to diagnose heart diseases. Zhang et al. [9] used wavelet energy and sub-smoothing filters to eliminate unwanted disturbances on ECG signals generated while recording the data. Chandra et al. [10] performed denoising techniques and feature extraction to detect arrhythmia. Median filter, Gaussian filter, and other filters were used by Subbiah et al. [11] to denoise the signal and identify which filter gives the best result. Kumar et al. [12] used multidimensional noise cancellation of ECG signal to extract pure ECG signal. Rajeshwari et al. [13] used efficient preprocessing techniques on MRI images. Pace et al. [14] developed interactive algorithm to segment heart chambers, epicardial surfaces and great vessel walls from pedic cardiac MRI of congenital heart diseases. Sraitih et al. [15] implemented different machine learning algorithms such as support vector machine (SVM) K-nearest neighbor(KNN) on the preprocessed ECG data for classifying different types of beats. Berntson et al. [16] used a high-pass filter to measure root mean square successive heart period variability. Ali et al. [17] used different deep learning architectures, such as convolutional neural networks (CNNs), long short-term memory (LSTM), autoencoders, etc., to classify ECG signals to detect arrhythmia. Naz et al. [18] took ECG data as an input, convert the data into a binary image, classify using deep learning, and discussed the limitations of using ECG data rather than image data. Wu et al. [19] used convolutional neural networks on a denoised ECG signal to classify different peaks in the signal. Aziz et al. [20] used two-event corresponding moving average (TERMA) and fractional Fourier transform (FrFT) to extract different peaks and then used machine learning to classify the heart as normal or abnormal.

Pattern detection and feature extraction from the raw data produce multi-layered long-duration crucial in the whole process. Patro et al. [21] used ANN on ECG data to identify patterns based on psychological and geometrical conditions of the heart used for biometric identification. Lastre et al. [22] identified heart abnormalities after FIR smoothing on data. Acharya et al. [23] implemented grayscale feature extraction from electrocardiographic images for identifying CAD using the GMM classifier. Extraction of heart rate signals from ECG to identify CAD by using linear and nonlinear analysis was performed by Acharya et al. [24]. Bhyri et al. [25] used CNN for feature extraction from ECG to identify QRS complexes in the data. Lin et al. [26] efficiently implemented the use of convolutional neural networks for feature extraction using a person’s facial features, which indeed helps identify coronary heart diseases. ML algorithms were used by Akella et al. [27] for feature extraction and classification of heart diseases. Valluraiah et al. [28] identified and located R peaks and QRS detection using the Hilbert transform.

The last phase would identify and classify arrhythmia and heart diseases using the preprocessed data4 and extracted features. Yıldırım et al. [29] used 1-dimensional CNN to identify cardiac arrhythmia with the help of long-duration ECG signals. This method achieved 91% accuracy and took much less time when compared with traditional methods.

Luz et al. [30] performed both preprocessing of ECG data and classification of two significant types of arrhythmias using the preprocessed data. A relevance vector machine (RVM) is used to classify five types of arrhythmias which produced more significant results than Gayathri et al. [31]. Rajpurkar et al. [32] used 34-layered convolutional neural networks to identify arrhythmia using ECG signals. ECG signals are converted into 2-D vectors by Li et al. [33] to detect irregular heartbeats to identify abnormalities that have produced more significant results. Avanzato et al. [34] used multi-layered CNN onto the ECG dataset to classify CAD, and it produced an accuracy of ~98%. Alizadehsani et al. [35] used different ML algorithms to analyze factors that cause CAD and thus detect CAD. Acharya et al. [36] and Acharya et al. [37] used multi-layered CNN for analyzing long-duration ECG signals for detecting CHD. The detailed overview of models, datasets, and their accuracy in diagnosis of heart related diseases are presented in Table 1. 

## 3. Methodology

This section is as follows. Section 3.1 briefly describing the dataset and its contents. Section 3.2 contains preprocessing information about the data. Section 3.3 explains about different filters applied to ECG signals. Section 3.4 describes the model architecture, which is further explained in Section 3.4.1, Section 3.4.2 and Section 3.4.3 about model 1, model 2, and model 3, respectively. Finally, Section 3.5 explains performance metrics used to evaluate and measure the model’s performance.

### 3.1. Data Description

**Figure 1:**
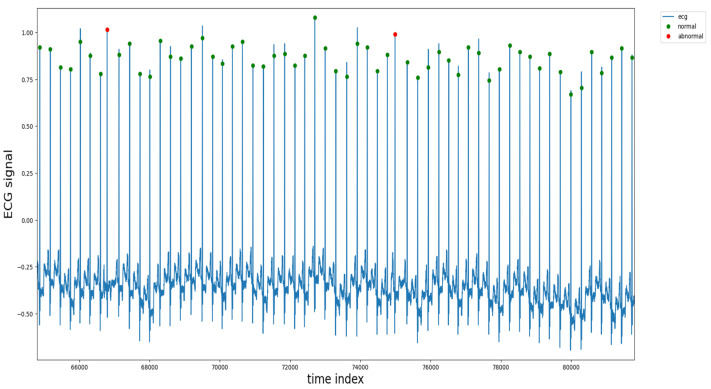
Annotations of heartbeats in the dataset.

Forty-eight half-hour labeled two-channel ambulatory ECG recordings were presented in the MIT-BIH Arrhythmia Database. In the dataset above, 47 people were investigated between 1975 and 1979 in the BIH arrhythmia laboratory [38] and published in 2005. A random selection of twenty-three recordings was made from a collection of 4000 24-h ambulatory ECG recordings collected from a diverse population of outpatients (40%) and inpatients (60%) at Boston’s Beth Israel Hospital. The remaining 25 recordings were selected from the same set to include rare but clinically significant arrhythmias. This was done to ensure the dataset is generalized and contains a variety of arrhythmias. The ECG recording of the database was converted to digital format, and two cardiologists resolved any difference present in data. The annotation of heartbeats is presented in Figure 1.

### 3.2. Preprocessing

The ECG signal from MIT-BIH Arrhythmia Database was sampled at 360 Hz. The data from the dataset are well-curated, but in the real-world scenario, ECG signals contain noise, so to test and train the proposed model to perform under those circumstances, the random noise was added to the dataset. The denoising of the dataset was performed using wavelet transformation, median filter, 1-D Gaussian filter, Moving Average filter, Savitzky–Golay filter, and low-pass Butterworth filter. The performance of the filters is presented in the result section, out of which the median filter performed well compared to other filters; therefore, we used it to denoise the ECG signal. ECG signals are segmented into heartbeats as per the annotation provided in the dataset. A Z-score normalization procedure normalizes each ECG segment to increase the data and reduce noise.

This procedure overcomes the problem of amplitude scaling and removes the offset effect. After preprocessing, the data in the dataset are first randomized, then divided into training sets and testing sets with 80% and 20% of total data, respectively. Eighty percent of training data is fed to the model to train on the data, and then the 20% test data is used to test and evaluate the model.

### 3.3. Filters

Noises are unwanted signals in data acquisition that must be denoised for processing signals for critical situations. The authors used six filters to denoise and filter out the noise from the ECG data. The filters are as follows:

1. Median Filter:

The median denoising filter filters out salt and pepper type noise [39]. It is a nonlinear filter. In this filter, a window slides over the input, and in each case, the median of the window is calculated, and the median value replaces other pixels.

2. Gaussian Filter:

A Gaussian denoising filter is also known as a Gaussian blur. It denoises the signal and creates kernels with normal distribution [40]. The window size is restricted to a value in this filter as Gaussian filters generally use an infinite range for each input. A Gaussian impulse has been created that denoises the entire input data or signal.

3. Moving Average Filter:

Smoothing the signal is one of the main tasks performed by the Moving Average filter. This filter finds the average over the data points on the whole input signal to smoothen the signal [41]. This filter smoothens short-term fluctuations and disturbances to observe long-term trends in the signal.

4. Savitzky–Golay filter:

In this filter, some points to fit a polynomial and replace the input with the output, which smoothens the signal on performing this process over the signal input [42,43].

5. Low-Pass Butter Filter:

It is designed to make frequency response as flat as possible on the passband. It allows the input signal to appear at the output until the frequency is lower than the cutoff frequency. It is always assumed to make the signal smooth and keep the frequency low [44].

6. Wavelet Denoising Filter:

Wavelet consists of oscillations where the oscillation begins at 0, increases, and decreases. This filter filters the signal in the wavelet space using the threshold value then inverts the filtered signal to produce the original [43].

### 3.4. Architecture

This paper presents three (1D) custom CCNN out of which two 1-dimensional (1D) custom CCNN structures (Model 1, Model 2) consisting of five (5) convolution layers, five (5) max-pooling layers, and one (1) fully connected layer. The third custom CCNN (Model-3) consists of four (4) convolution layers, four (4) and three (3) fully connected layers, max-pooling layers convolution layer, dropout, and max-pooling alternate each other in the models.

#### 3.4.1. Model 1

**Figure 2:**
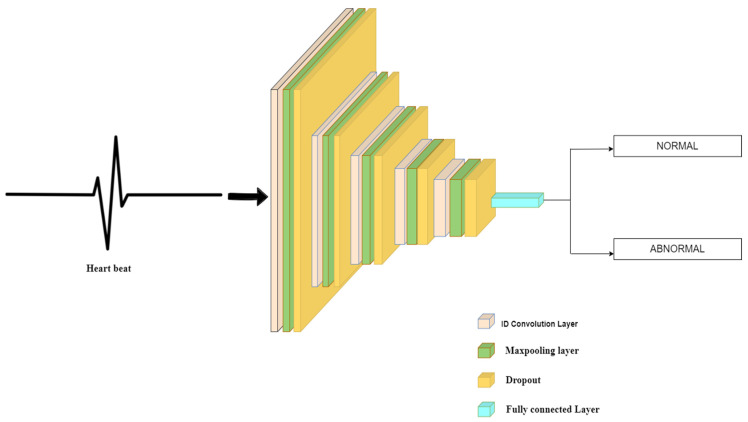
Architecture diagram of Model-1.

In model 1, as shown in Figure 2, the input shape is (2160, 1), and each layer has RELU as its activation function. Each alternating convolution layer has filters as 400, 256, 178, 88, and 44, respectively, and kernel sizes of 20, 15, 7, 5, and 3. The dropout rate between the first three sets of convolution and max pool layers is 0.5, but it is set to 0.25 for the last two sets. Max pooling layers are set to pool size as 2, consisting of strides which are also set to 2, and padding is present. The final layer, the output layer, has an activation function as a sigmoid.

#### 3.4.2. Model 2

**Figure 3:**
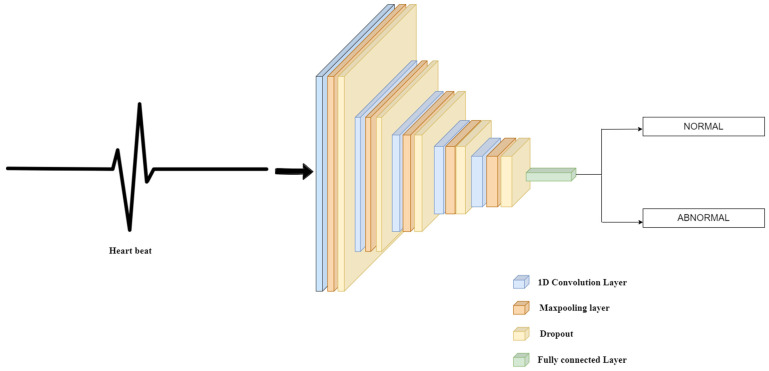
Architecture diagram of Model-2.

In Model 2 presented in Figure 3, the structure is as follows: a dropout separates the convolution and max-pooling layers. The input layer has an input size of (2160, 1). The convolution layers, including the input layer, have an activation function as RELU. The filters of alternating convolution layers are set to 600, 400, 266, 178, and 88, respectively, and the kernel size of each alternating layer is 20, 15, 10, 7, and 20, respectively. Each convolution layer and max-pooling layer set has a dropout place between them, with a rate of 0.25 throughout the model. Each max pool layer has padding activated, has a max pool size of two (2), and strides set to two (2). The model is flattened before the dense layer, and the dense layer has activation specified as sigmoid.

#### 3.4.3. Model 3

**Figure 4:**
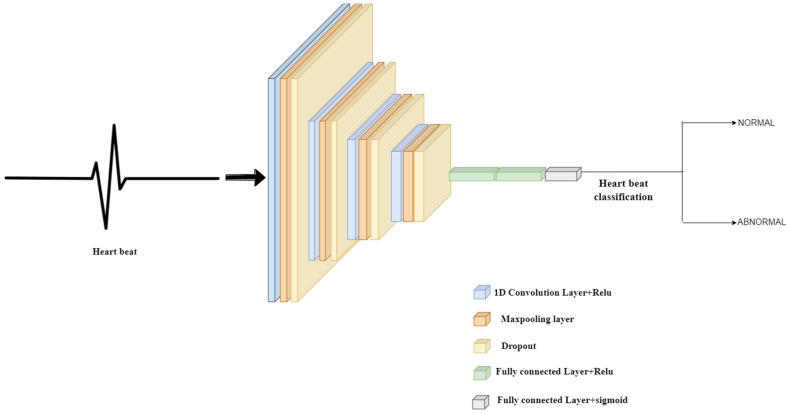
Architecture diagram of Model 3.

The structure of Model-3 shown in Figure 4 is as follows: a dropout separates the convolution layer and max-pooling layer. The input layer has an input size of (2160, 1). The convolution layers, including the input layer, have an activation function as relu. The filters of alternating convolution layers are set to 256, 128, 72, and 36, respectively, and the kernel size of each layer is 5. Each convolution layer and max-pooling layer set has a dropout place between them, with a rate of 0.50 throughout the model. Each max pool layer has padding activated, has a max pool size of two (2), and strides set to two (2). Before the dense layers, the model is flattened. Three dense layers are placed with neurons (50, 32, 1), activation function used in the first two dense layers is relu, and the activation function specified in the third layer is sigmoid.

### 3.5. Performance Matrix

The filters are compared using peak to signal noise ratio (PSNR), which is defined as the ratio between the maximum value of the signal to the distorting noise in the signal.

(1) $$\mathrm{PSNR}=10\log_{10}(\frac{{\left(L-1\right)}^{2}}{MSE})\;=20\log_{10}(\frac{\left(L-1\right)}{RMSE})$$

where *MSE* is mean square error, *RMSE* is root mean squared error, and *L* represents number of maximum possible intensity levels.

Evaluation of a model is one of the essential steps in building a neural network. Evaluation of a model focuses on testing the model’s performance on the test dataset after training the model. ECG signal classification performance measure is done using loss, accuracy, sensitivity, specificity, precision, and recall.

The confusion matrix, the N × N matrix, where N represents numbers of classes in classification, is one of the critical elements in the performance metric. It is plotted as follows.
True positive (TP)False Positive (FP)True Negative (TN)False Negative (FN)

Here, true positive represents that the classified data point is positive and classified as positive by the model. True negative represents that the model’s classified data point is negative and is classified as negative by the model. False positive represents that the classified data point is negative and is classified as positive by the model. False negative represents that the classified data point is positive and is classified as negative by the model.

Accuracy = (TP + TN)/(TP + FP + FN + TN)(2)

Sensitivity = TP/(TP + FN)(3)

Specificity = TN/(TN + FP)(4)

precision = TP/(TP + FP)(5)

Recall = TP/(TP + FN)(6)

**Figure 5:**
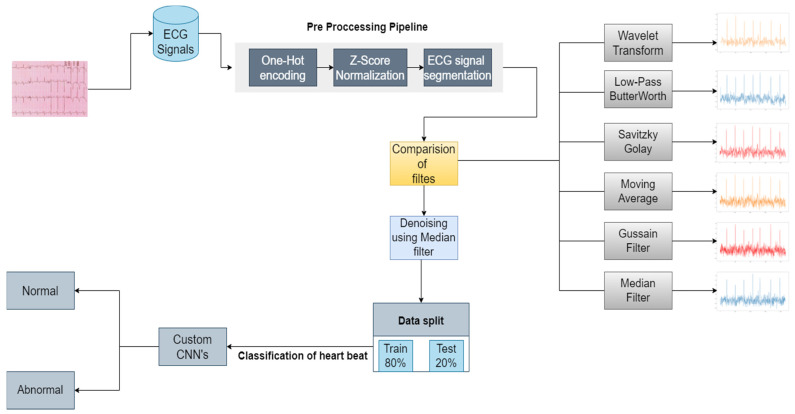
Proposed methodology for the classification of a heartbeat using custom CCNNs.

Figure 5 represents the process flow starting from the data and classifying it.

ECG signal is taken from the dataset on which one-hot encoding is performed. These data are normalized using Z-score, and then heartbeat segmentation is done. The data are denoised using different filters such as wavelet transform, low-pass Butterworth filter, Savitzky–Golay filter, moving average filter, median filter, and gaussian filter. The preprocessed and denoised data is now split into training and testing sets and then fed to CCNNs to perform the heartbeat classification.

## 4. Results and Discussion

This part of the paper as follows: Section 4.1 provides details about the filters used in the study, and their performance is analyzed on the dataset. Comparative analyses of the filters are done in Section 4.1.7 using peak to signal noise ratio (PSNR); Section 4.2 explains and presents the results of three different convolutional neural networks used in this experimental study.

### 4.1. Denoising

Denoising is a process in which signals are reconstructed and extracted from noisy and mixed signals. Its main goal is to eliminate noise and preserve helpful information. Some denoising techniques are discussed, and their results are presented in this section.

#### 4.1.1. Wavelet Denoising Filter

**Figure 6:**
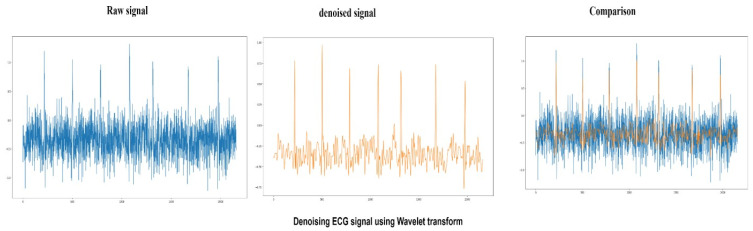
Raw ECG signal (Blue) denoised using wavelet transform and comparison between denoised signal (Orange) and raw signal.

Wavelet denoising depends on the wavelet representation of the signals. Small values in the wavelet domain are Gaussian noise which can be removed by setting coefficients below threshold or zero, or all coefficients are shrunk toward zero by the given amount. This study used soft thresholding, Bayes Shrink algorithm, and sym8 wavelet in denoising raw signals. It achieved a peak signal-to-noise ratio of 56.9, and the results of wavelet denoising can be seen in Figure 6.

#### 4.1.2. Median Filter

**Figure 7:**
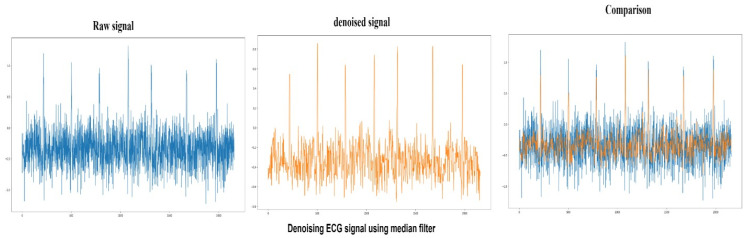
Raw ECG signal (Blue) denoising using median filter and comparison between denoised signal (Orange) and raw signal.

It is a nonlinear filtering technique often used to remove impulse noise from signals. Removal of noise from the raw signal is a preprocessing step. The central concept behind median filtering is to run signal entry by entry, replacing each entry with the median of the neighboring entries. The pattern of neighbors used to find the median is called a window, which slides entry by entry over the entire signal. The peak to signal noise achieved by the median filter in this study is 87.3, and the results of median filter denoising can be seen in Figure 7.

#### 4.1.3. Gaussian Filter

**Figure 8:**
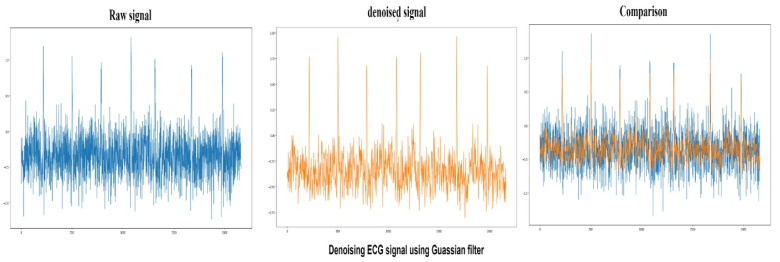
Raw ECG signal (Blue) denoising using Gaussian filter and comparison between denoised (Orange) and raw signals.

The Gaussian function is the impulse response of the Gaussian filter. The accurate gaussian response would have an infinite impulse response. Convolution of the input signal and Gaussian filter modify and denoise signals. The window size is restricted to a value in this filter as Gaussian filters generally use an infinite range for each input. A gaussian impulse is created, which denoises the entire input data or signal. In this study, the Gaussian filter achieved a peak to signal noise value is 86.5, and the output of the Gaussian filter can be seen in Figure 8.

#### 4.1.4. Moving Average Filter

**Figure 9:**
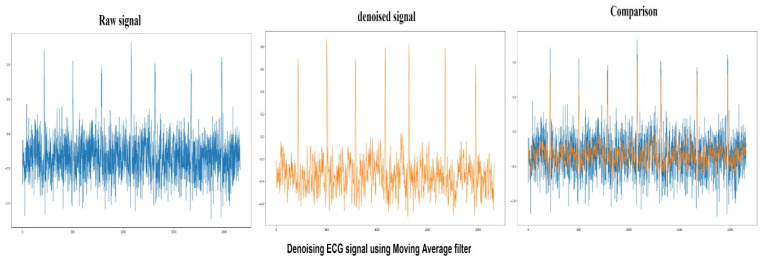
Raw ECG signal (Blue) denoising using Moving average filter and comparison between denoised (Orange) and raw signals.

A moving average filter is simply a low-pass Finite Impulse Response (FIR). This filter is used to regulate an array of sample data or signals. Samples of the input are taken at a time. Then, input samples and an average of those values are taken to signal output. It is found that as the length of the filter increases, the smoothness of the output increases. The peak to signal noise ratio achieved by moving the average filter is 81.05 and the denoised signal produced, and its comparison with a raw signal is presented in Figure 9.

#### 4.1.5. Savitzky–Golay Filter

**Figure 10:**
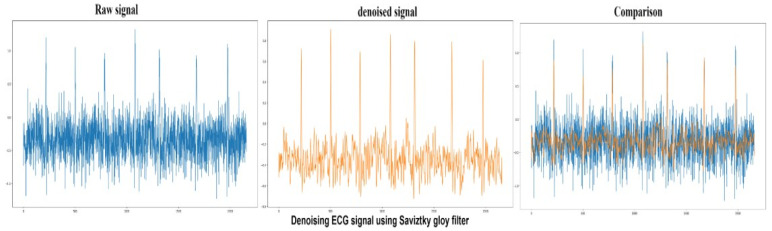
Raw ECG signal (Blue) denoising using Saviztky Golay filter and comparing denoised (Orange) and raw signals.

Savitzky–Golay filters are commonly used to remove signals whose frequency span is significant. Savitzky–Golay filters are also known as digital smoothing polynomial filters or least-squares smoothing filters. These filters perform better than standard averaging FIR filters in some applications, which filters high-frequency signals with noise. Savitzky–Golay filters are more successful at preserving high-frequency signals. The peak to signal noise ratio achieved by the Savitzky–Golay filter is 80.5 in this study, and the filter is implemented using Scipy library with window size 25 and polynomial of order 7 is used to fit the sample. The performance of the Savitzky–Golay filter can be seen in Figure 10.

#### 4.1.6. Low-Pass Butterworth Filter

**Figure 11:**
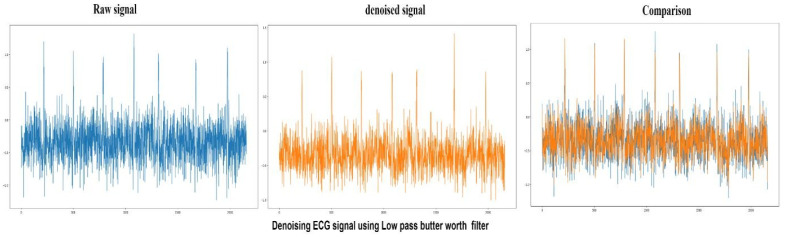
Raw ECG signal (Blue) denoising using low-pass Butterworth filter compares denoised (Orange) and raw signals.

Butterworth filters are those filters whose frequency is flat after the passband region. The output provided by the low pass filter from DC up to a cut-off frequency f(H) and the signal above the frequency gets rejected by the low-pass Butterworth filter. The peak to signal noise ratio achieved by the low-pass Butterworth filter is 78.6 in this study, and the performance of the low-pass Butterworth filter is shown in Figure 11.

#### 4.1.7. Comparison between Filters

The details of the ECG signals of the MIT-BIH dataset are present in the three files. They are (.hea) denoted as a header file, (.dat) denoted as a binary file, and (.atr) denoted as a binary annotation file.

The header file holds comprehensive information about the ECG signal, such as lead used for the patient and the number of leads used to diagnose diseases, the sampling frequency of the signals, and patient details. Format of the signal is present in the binary file, and information related to beats is stored in the binary file. Multiple filters are implemented on ECG signals before segmenting them into the single-single heartbeats to remove different kinds of noises like muscle artifact noise, electrode motion artifact noise, and baseline wander. A comparison of the outputs of the filters was made using PSNR.

It can be observed from Table 2 that median and Gaussian filters perform better than other filters in removing noise from raw ECG signals.

### 4.2. Results of CCNNs

This experimental study was conducted on Google colab with Tesla K80 GPU, CPU Intel(R) Xenon(R), RAM 13 Gb. We use the proposed two convolutional neural networks and one Residual neural network. These are trained using heartbeats segmented from the ECG signal database. The results achieved by the models are presented in Table 3 and Table 4. It can be seen from the tables that Model-1 achieved better results than the other two models.

### 4.3. AUC–ROC CURVE

Receiver operating characteristic curve (ROC) is a plot between two parameters—True Positive Rate (TPR) and False Positive Rate (FPR)—which are plotted on Y and X axis, respectively. ROC curve is plotted by computing TPR and FPR at different thresholds and plotting it onto the graph to find the best threshold for the model.

TPR = TP/(TP + FN)(7)

FPR = FP/(FP + TN)(8)

ROC plot shows the following:

- Relationship between sensitivity and specificity. As sensitivity increases specificity increases.
- The classification power of the model at different thresholds. As the threshold decreases more data items are classified as positive.
- Test accuracy which can be identified as the closer the curve to the top leftmost corner of the graph accurate the model is. An ideal curve would go straight from zero up to the top-left corner and then parallel to the X-axis. The curve which will be nearer to the diagonal would be less accurate.

Area Under Curve (AUC) is used to summarize the performance of the ROC curve. AUC curve is the measure of the ability of the model to distinguish between the classes.

**Figure 12:**
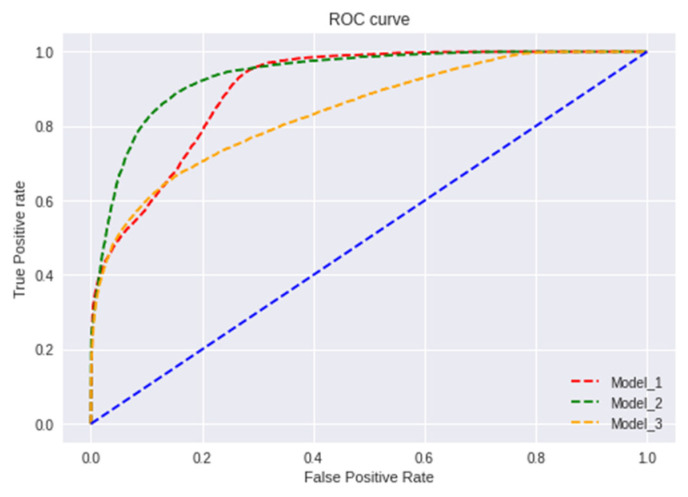
Receiver operating characteristic curve (ROC).

**Figure 13:**
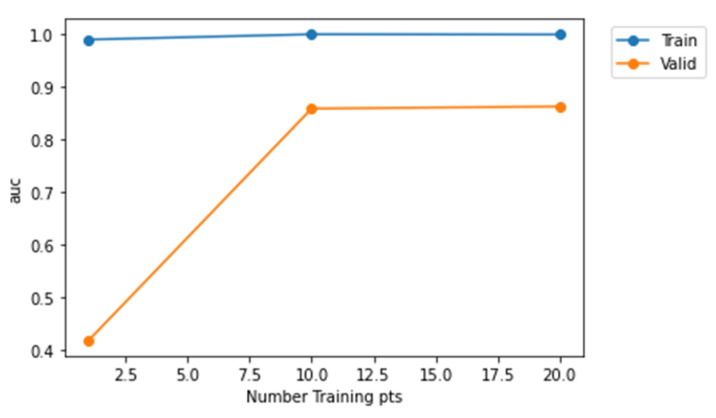
Representation of AUC values at different training points for Model-1.

**Figure 14:**
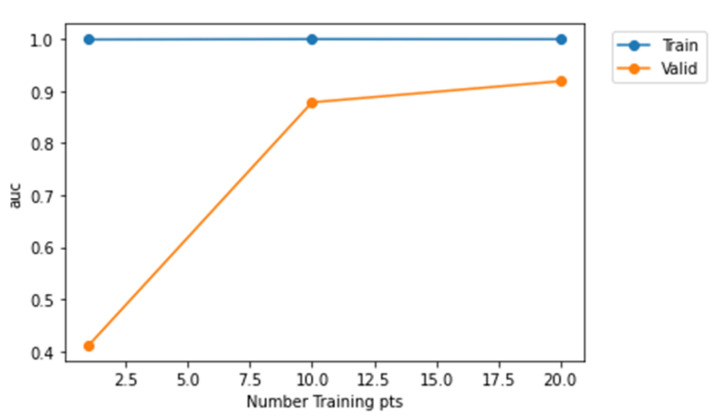
Representation of AUC values at different training points for Model-2.

**Figure 15:**
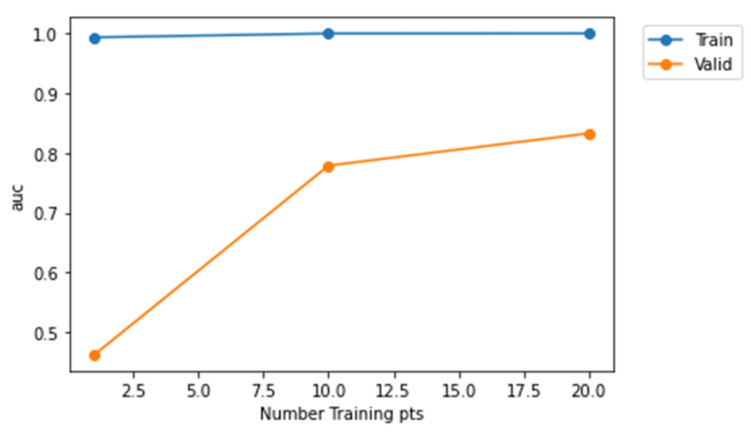
Representation of AUC values at different training points for Model-3.

From Figure 12, it is evident that Model-2 curve is much nearer to the top left corner and Model-3 is much nearer to the diagonal. Therefore, Model-2 has a higher AUC value when compared to Model-1 and Model-3. Furthermore, Figure 13, Figure 14 and Figure 15 represent AUC values of the model at different number of training points. Model-2 at any instant has the higher AUC value when compared with Model-1 and Model-3. Therefore, Model-2 has higher performance than Model-1 and Model-3 when the models are compared using ROC curve that is shown in Figure 12 and AUC values.

### 4.4. Confusion Matrix

**Figure 16:**
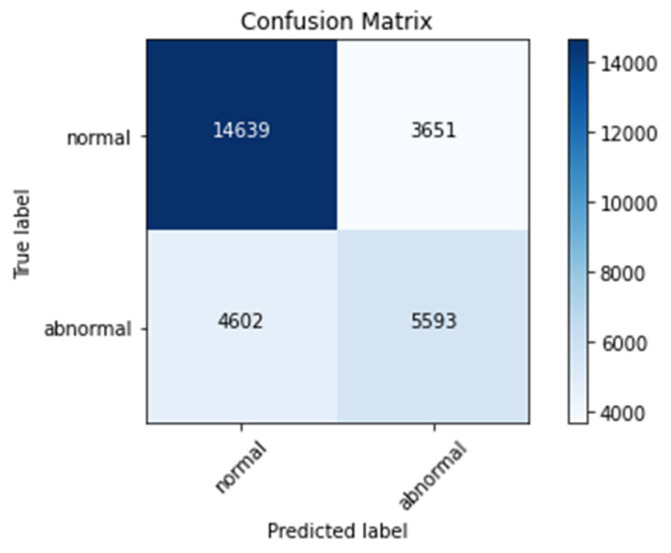
Confusion matrix for Model-1.

**Figure 17:**
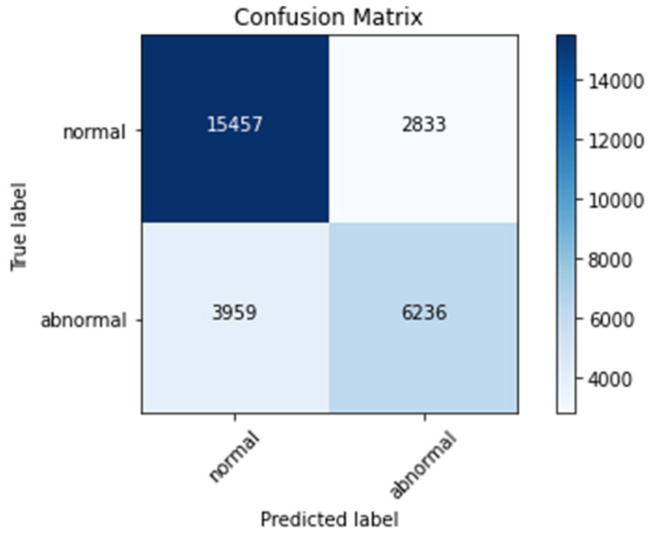
Confusion matrix for Model-2.

**Figure 18:**
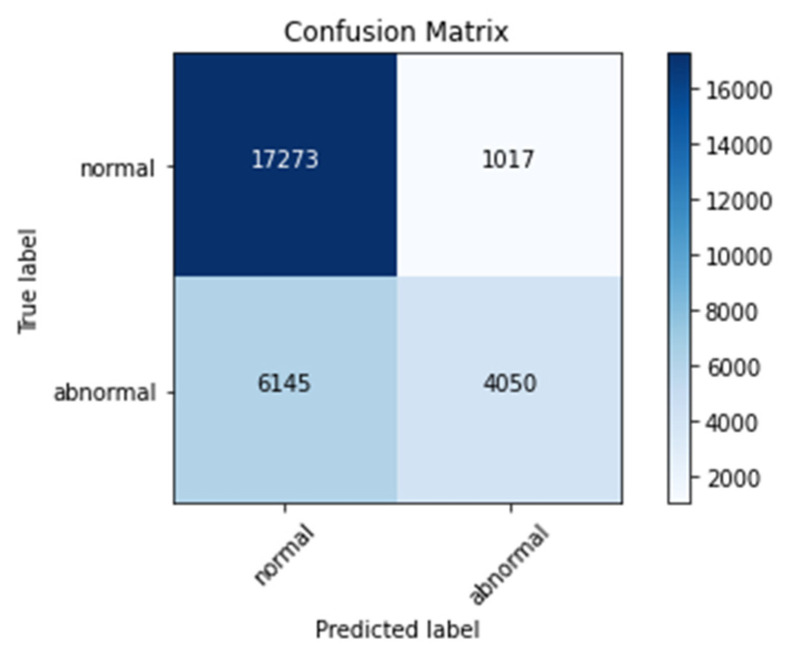
Confusion matrix for Model-3.

Figure 16, Figure 17 and Figure 18 represent confusion matrix of model-1, model-2, and model-3, respectively. Confusion matrix is used to obtain parameters of evaluation such as accuracy, sensitivity, specificity, precession, and recall.

From Table 4, it can be noted that validation accuracies are 86%, 87%, and 93% for Model-3, Model-1, and Model-2, respectively. These values implies that Model-2 had performed well compared to other two models on the fed ECG data.

## 5. Discussion

The deep learning architectures Model-1, Model-2, and Model-3 proposed for ECG classification are motivated by classification and analysis [45,46]. Various studies are performed using conventional neural networks to characterize abnormal ECG signals. Our ECG monitoring and classification system, which is patient-specific, was developed using a three-layer convolutional neural network structure. R-wave was used to detect ventricular and supraventricular electrical activity in this system, giving 99.60% and 97.60% accuracy, respectively. In Zubain et al. [47], 3-layer convolutional neural network model was trained using R peak ECG beat patterns and achieved an accuracy of 92.7% in detecting five different ECG classes. Four ECG classes were characterized by an 11-layer convolutional neural network using two and five seconds of ECG signal. They yielded an accuracy of 92.50%, a sensitivity of 98.09%, and a specificity of 93.13 for two-second ECG. Furthermore, this system achieved 94.90% accuracy, 99.13% sensitivity, and 81.44% specificity for five seconds of ECG signals (Acharya et al. [48]). Robust features were extracted from ECG signals using alternative convolutional, pooling, and dropout layers. Then, features were linked to fully connected layers for ECG signal characterization. The results presented in Table 3 and Table 4 reveal that the proposed model achieves remarkable results. Moreover, the implementation of the proposed architecture is economical and needs light hardware because architecture needs only 1-dimensional convolutions.

We have trained the proposed models with six epochs with 32 batch size. We have considered the epoch value as 6 as if we try with higher epoch size models will start memorizing the ECG patterns, and, thus, may lead to overfitting problem. Model-1 consists of 1,956,651 trainable parameters and has an average epoch time of 401 s. Model-2 consists of 5,274,443 trainable parameters and has an average epoch time of 883 s. Model-3 consists of 462,167 trainable parameters and has an epoch time as 163 s. Thus, Model-2 achieves better average epoch computation time than other models. The overall time complexity is O(K·N·D·L). Here, K represents length of the filter. N is the length of the input. D is the depth of the filter. L defines the number of filters.

The advantages of proposed models are as follows:

1. The proposed CCNN model is robust.
2. There is no requirement for QRS detection.
3. CCNN structure consists of feature extraction, selection, and classification.
4. The proposed model is light on the computation side; it is cost-effective.

The limitations are as follows:

1. The training phase of CCNN is much higher.
2. A huge database is required to fulfill the training criteria.
3. CCNN required a fixed ECG signal; thus, ECG signal length must be fixed for both the training and testing phase.

## 6. Conclusions and Future Scope

The most common cause of heart attack is coronary artery disease (CAD). Despite significant technical developments, an automated diagnosis method that is both reliable and efficient is required for the early detection of CAD. Three custom CCNN structures (Model 1, Model 2, and Model 3) were built out of which first two models consisted of five convolutional layers, five max-pooling layers, and one fully connected layer, and the third Model-3 consists of four convolutional layers, four max-pooling layers, and three fully connected layers to detect two classes (regular and CAD). Model-1 achieved 93.03% accuracy, 52.18% sensitivity, and 84.45% specificity. While Model-2 achieved 89.03% accuracy, 47.92% sensitivity, and 95.88% specificity. Model-3 achieved 89.56% accuracy, 47.48% sensitivity, and 87.20% specificity. The new technique can aid clinicians in accurately diagnosing coronary artery disease.

The approach is easy to use, inexpensive, and suitable for cardiac screening in developing countries. The scientists can use an extensive database to improve the CCNN structure in future research. This research can also be applied to the early detection of coronary artery disease (CAD), different phases of myocardial infarction (MI), and congestive heart failure (CHF) utilizing ECG signals. This will aid medics in providing appropriate medication and saving lives.

## Figures and Tables

**Table 1:** Summary of literature review.

Author	Models	Disease	Datasets	Accuracy
Altan et al. [8]	Deep belief networks	Coronary artery disease	Made a dataset from collecting data	98.88%
Ali et al. [17]	CNN, LSTM, RNN	Arrythmia classification	Combination of different publicly available datasets	-
Naz et al. [18]	Pretrained CNNs	ECG classification	MIT-BIH database	91.2
Wu et al. [19]	Convolutional neural networks	Arrhythmia	MIT-BIH database	97.41
Patro et al. [21]	Artificial neural network	Feature extraction from ECG signals.	MIT-BIH ECG ID database signal	-
Acharya et al. [23]	Gaussian Mixture Model (GMM)	Coronary artery disease	The CAD datasets from the University California Irvine a database	95%
Acharya et al. [24]	Convolution neural network	Coronary artery disease	Physio net databases	95.11%
Bhyri et al. [25]		heart diseases	CSE ECG database	around 99%
Lin et al. [26]	Deep convolutional neural networks	coronary artery disease	Combination of datasets	95%
Akella et al. [27]	SVM, K-NN, artificial neural network	coronary artery disease	UCI dataset	93.03%
Yıldırım et al. [29]	16-layer standard CNN	Arrhythmia	MIT-BIH Arrhythmia database	86.67%
Luz et al. [30]		Arrhythmia	MIT-BIH, EDB, AHA, CU, NST databases	-
Gayathri et al. [31]	Relevance vector machine	Arrhythmia	MIT/BIH database	RVM boosts generalization capability
Rajpurkar et al. [32]	34-layer convolutional neural network	Arrhythmia	Own dataset with a combination of datasets	
Li et al. [33]	CNN-based classification on ECG signals.	ECG classification	MIT-BIH arrhythmia database,	99.1%
Avanzato et al. [34]	Convolutional neural networks	coronary artery disease	MIT-BIH arrhythmia database	98.33%
Alizadehsani et al. [35]	ML algorithms	Coronary artery disease	Combination of different datasets	-
Acharya et al. [36]	11-layer deep convolutional neural network	congestive heart failure	BIDMC: Congestive Heart Failure Database, Fantasia Database, MIT-BIH database	99.01%
Acharya et al. [37]	Time level and frequency domain analysis	Coronary artery disease	CAD dataset	96.8

**Table 2:** Comparison of the filters.

Filters	Wavelet Transform	Low-Pass Butterworth Filter	Savitzky–Golay Filter	Moving Average	Gaussian Filter	Median Filter
PSNR	56.9	78.6	80.5	81.05	86.5	87.3

**Table 3:** Training results of the models used in the study.

Model	Training Loss	Training Accuracy	Training Sensitivity	Training Specificity	Training Recall	Training Precision	Training F_1_-Score
Model-3	0.0533	0.9829	0.9598	0.9933	0.9598	0.9853	0.9708
Model-1	0.0373	0.9888	0.9771	0.9942	0.9771	0.9872	0.9762
Model-2	0.0357	0.9907	0.9824	0.9946	0.9824	0.9890	0.9848

**Table 4:** Validation results of the models used in the study.

Model	Validation Loss	Validation Accuracy	Validation Sensitivity	Validation Specificity	Validation Recall	Validation Precision	Validation F_1_-Score
Model-3	0.3831	0.8671	0.4081	0.8250	0.3888	0.4351	0.3833
Model-1	0.3171	0.8737	0.4525	0.8502	0.4030	0.4438	0.3859
Model-2	0.2754	0.9325	0.4214	0.8625	0.4214	0.5207	0.4338

## Data Availability

MIT-BIH Arrhythmia dataset used in this research to train three custom convolutional neural networks and validate results. The authors confirmed that this dataset is available publicly in the physionet database.
